# PD-1 knockout on cytotoxic primary murine CD8^+^ T cells improves their motility in retrovirus infected mice

**DOI:** 10.3389/fimmu.2024.1338218

**Published:** 2024-04-29

**Authors:** Daniela Mittermüller, Lucas Otto, Annika Loredana Kilian, Ann-Kathrin Schnormeier, Elisabeth Littwitz-Salomon, Anja Hasenberg, Ulf Dittmer, Matthias Gunzer

**Affiliations:** ^1^ Institute for Virology, University Hospital Essen, University of Duisburg-Essen, Essen, Germany; ^2^ Institute for Experimental Immunology and Imaging, University Hospital Essen, University of Duisburg-Essen, Essen, Germany; ^3^ Institute of Cell Biology (Cancer Research), University Hospital Essen, University of Duisburg-Essen, Essen, Germany; ^4^ Institute for the Research on HIV and AIDS-Associated Diseases, University Hospital Essen, University Duisburg-Essen, Essen, Germany; ^5^ Leibniz-Institut für Analytische Wissenschaften - ISAS-e.V., Dortmund, Germany

**Keywords:** cytotoxic T lymphocytes, cell motility, anti-viral response, retrovirus, CRISPR/Cas9, PD-1, gene editing

## Abstract

Cytotoxic T lymphocyte (CTL) motility is an important feature of effective CTL responses and is impaired when CTLs become exhausted, e.g. during chronic retroviral infections. A prominent T cell exhaustion marker is programmed cell death protein 1 (PD-1) and antibodies against the interaction of PD-1 and PD-ligand 1 (PD-L1) are known to improve CTL functions. However, antibody blockade affects all PD-1/PD-L1-expressing cell types, thus, the observed effects cannot be attributed selectively to CTLs. To overcome this problem, we performed CRISPR/Cas9 based knockout of the PD-1 coding gene *PDCD1* in naïve Friend Retrovirus (FV)-specific CTLs. We transferred 1,000 of these cells into mice where they proliferated upon FV-infection. Using intravital two-photon microscopy we visualized CTL motility in the bone marrow and evaluated cytotoxic molecule expression by flow cytometry. Knockout of *PDCD1* improved the CTL motility at 14 days post infection and enhanced the expression of cytotoxicity markers. Our data show the potential of genetic tuning of naive antiviral CTLs and might be relevant for future designs of improved T cell-mediated therapies.

## Introduction

Cytotoxic T lymphocytes (CTLs) play a key role in the host immune response against virus infections including retroviruses like Human Immunodeficiency Virus (HIV) (1–4). Their function is to actively migrate and search for virus-infected cells for their elimination through the release of lytic granules (5–7). However, in the course of a persistent infection, CTLs can become exhausted, leading to inefficient virus elimination and the establishment of chronic infection (8, 9). T cell exhaustion affects the immune response against various diseases, including virus infections and many forms of cancer (10). A murine model to study the immune response throughout a chronic retroviral infection is the Friend virus (FV) model. Infection of C57BL/6 mice is controlled during the acute phase of the infection but the virus cannot be completely eliminated, resulting in T cell exhaustion and viral chronicity. Studies in the FV model paved the way to a better understanding of the immune response in chronic virus infections and hence FV is a valuable tool to investigate T cell exhaustion (11).

Understanding T cell exhaustion and finding ways to reactivate CTLs from this state poses a highly relevant goal of medical research. One aspect of T cell exhaustion receiving little attention thus far, is the motility of CTLs. The anti-viral response of CTLs is accompanied by excessive motility of the individual CTLs, which they need to effectively search for and find target cells (6, 12, 13). However, CTLs have been shown to lose much of their motility during the development of T cell exhaustion (12, 14). This loss of motility contributes to inefficient virus elimination (12). Modulating CTL motility might, therefore, be a target of interest to improve CTL responses.

One of the most studied molecular interactions in T cell exhaustion focuses on the interaction of the immune checkpoint molecules programmed cell death protein 1 (PD-1) and its ligand PD-L1. Previous studies in Lymphocytic Choriomeningitis Virus (LCMV) showed that blocking the PD-1-PD-L1 axis restores effective CTL responses (9). PD-1 expression on T cells is increased at day 10 post FV infection (15) and immune checkpoint therapy, which included PD-L1 blocking antibodies, has also been shown to improve CTL function and FV control in chronic infection (16). In addition, blocking the PD-1-PD-L1 axis increased CTL motility in persistent LCMV infection (14). This interesting study highlights a possible impact of the PD-1-PD-L1 axis on CTL motility. However, blocking antibodies affect all cells that express PD-1 and secondary effects of this treatment can therefore not be ruled out. Hence, we performed a CRISPR/Cas9 mediated knockout (KO) of the PD-1 coding gene *PDCD1* on naïve primary CD8+ T cells that are specific for an immunodominant FV epitope (17). We used our established two-photon intravital bone marrow imaging model (12, 18) to evaluate the impact of cell-selective PD-1 knockout on CTL motility in the bone morrow of living FV-infected mice. Bone morrow was chosen because it is an organ of massive viral replication during acute FV-infection (19). Our results contribute to a better understanding of the factors that influence CTL motility and might be relevant for future genetic editing of CTLs for T-cell therapy.

## Methods

### Mice

Experiments were performed using female and male C57BL/6 (C57BL/6JOlaHsd, Envigo, Horst, Netherlands) and DEREG-transgenic C57BL/6 mice (min. 8 weeks old) expressing a simian diphtheria toxin (DT) receptor–enhanced GFP (DTR-EGFP) fusion protein under the control of the endogenous forkhead box P3 (*Foxp3*) promoter/enhancer regions on the BAC transgene as recipient mice (20). DEREG mice were originally provided by Tim Sparwasser and bred at the University of Duisburg-Essen and the University Hospital Essen under pathogen-restricted conditions. For donor mice we used the previously described TCR-Lck-tdTom mice, which show a T cell specific expression of tdTomato (tdTom) as well as a FV-specific TCR directed against the GagL^85-93^ -epitope on more than 90% of all CD8^+^ T cells (17, 18).

All animal experiments were reviewed by the central animal laboratory (ZTL) and office for nature, environment and consumer protection of North-Rhine Westphalia (LANUV) and conducted in accordance with the regulations of the local animal welfare. Mice were kept at pathogen-restricted conditions and handled in accordance with institutional guidelines.

### Virus and viral infection

For infection we used FV stocks, which contained a complex of B-tropic Friend murine leukemia helper virus (F-MuLV) and spleen-focus forming virus (SFFV). The virus stocks were prepared as previously described (12, 21). Recipient mice were infected by intravenous injection of 20,000 spleen focus-forming units of FV in 100 µL PBS. The virus stocks were free of lactate dehydrogenase-elevating virus (22).

### Cell isolation and adoptive cell transfer

Blood draw from donor mice, CD8 T cell isolation and preparation for cell transfer was carried out as previously described (12, 18). For transfer of *PDCD1* targeted and control CD8+ cells, gene editing was performed as described below prior to cell transfer. 1,000 purified cells were transferred intravenously, suspended in 100 µL PBS, into recipient mice approximately 4h after FV-infection.

### CRISPR/Cas 9 based gene editing

The Lonza P3 primary cell 4D-Nucleofection Kit (Lonza, Basel, Switzerland) based nucleofection protocol was adapted to the previously published protocol by Nüssing et al. (23). P3 buffer was freshly prepared for each experiment by mixing 3.6 µl Supplement 1 to 16.4 µL Primary Cell Solution per sample and kept at the 4°C until shortly before use. Three different gRNA complexes (24) were formed from *PDCD1* crRNAs AA-AC (IDT, Coralville, IA, USA, Design ID: Mm.Cas9.PDCD1.1.AA - AC) and tracrRNA (IDT, cat. No. 1072533), respectively. Complex was formed by equimolar mix of *PDCD1* crRNA with tracrRNA and incubation for 5 minutes at 95°C followed by letting the formed gRNA complexes cool down to room temperature for up to three minutes. 1 µL of each formed gRNA complex was pooled in one tube and 0.6 µL Cas9 protein (IDT, cat. No. 1081059) was added, topped up with 0.4 µL RNase free water and mixed, briefly spun down and incubated for 10 min at room temperature for RNP formation. For control nuclefection, unspecific negative ctrl crRNA (IDT, cat. No. 1072544) was used for gRNA complex formation and subsequent RNP formation. After incubation 1 µL electroporation enhancer (IDT, cat. No. 1075916) was added to the solution and isolated CD8+ T cells were resuspended in 20 µL freshly prepared P3 buffer, added to the 5 µL gRNA/Cas9-RNP mix and transferred to the bottom hole of a well of the Lonza nucleofector strip. Nucleofection was performed using the DN100 puls of the 4D-Nucleofector system (Lonza) in the Institute for Cell Biology (Cancer research) in the University Hospital Essen. Cells were transferred to 175 µL 37°C pre-warmed RPMI medium (Thermo-Fisher Scientific, Waltham, MA, USA) supplemented with 10% FCS (Thermo Fisher Scientific), 100 U/mL Penicillin and Streptavidin (Sigma-Aldrich), 1x non-essential amino acids (Thermo Fisher Scientific), 2 mM L-glutamine (Thermo Fisher Scientific), 10 mM 2-(4-(2-Hydroxyethyl)-1-piperazinyl)-ethanesulfonic acid (HEPES) (Sigma-Aldrich), 1 mM sodium pyruvate (Thermo Fisher Scientific) and 50 µM β-mercaptoethanol (Thermo Fisher Scientifc) (T cell medium). Cells were stored in a cell culture incubator (5% CO_2_, 37°C) until preparation for cell transfer or preparation of in-vitro KO validation. Datasets were excluded from analysis upon insufficient PD-1 KO efficiency.

### In-vitro KO validation

Nunc MaxiSorp 96-well flat bottom plates (Invitrogen, Carlsbad, CA, USA) were coated with CD3e monoclonal antibody (eBioscience, San Diego, CA USA, cat No.16-0031-85) the day before nucleofection by adding 100 µL NaCO_3_ containing 10 µg/mL antibody and incubated at 4°C overnight. Before cell seeding, wells were carefully washed with PBS two times. For in-vitro KO validation up to 6 x 10^5^ nucleofected cells or 10^5^ unnucleofected CD8+ T cells were re-suspended in T cell medium supplemented with 1 µg/mL CD28 monoclonal antibody (eBioscience, cat. No. 14-0281-82) and seeded into anti-CD3e pre-coated well.

### Infectious centre assay

Serial dilutions of isolated bone marrow cells were seeded on *Mus dunni* cells and incubated at 37°C and 5% CO_2_ for 3 days. Cells were fixed with 96% Ethanol followed by staining with F-MuLV envelope-specific mAb 720 (25). Subsequently cells were stained with peroxidase-conjugated goat anti mouse IgG Ab (Sigma-Aldrich, St. Louis, MO, USA) and an aminoethylcarbazol (Sigma-Aldrich) substrate to visualize foci that originated from infected cells.

### Intravital two-photon microscopy and movie analysis

Intravital two-photon microscopy was carried out as previously described (12, 18, 26–28). Two-photon microscopy was done using a Leica TCS SP8 MP microscope (Leica Microsystems, Mannheim, Germany) with HCX IRAPO L25×/0.95-NA water-immersion objective, two external hybrid reflected-light detectors (HyD), and two external photomultiplier tubes (PMT). Imaging was performed with a titanium-sapphire laser (Coherent Cameleon Vision II, Santa Clara, CA, USA) tuned to 950 nm for intravital microscopy. FV-specific tdTom+ CTLs (PMT, 585/40 filter) and solid bone visualized by second-harmonic-generation (SHG) signal (HyD, 460/50 filter) were detected. For videos, one z-stack of up to 227.86 µm per minute or less time with a maximum step size of 3 µm, an imaging speed of 400 Hz and a pixel size of 1.16 was recorded in a format of a minimum of 590.48 µm x 590.48 µm for a collective video time of up to 30 minutes. Videos were recorded in the LAS X software (Leica Microsystems Mannheim, Germany).

Movie analysis was carried out using IMARIS version 9 and 10 as previously described (12). Movies were excluded from analysis if no cell motility was observable or KO validation showed insufficient KO efficiency.

### Flow cytometry

Antibodies used for cell surface and intracellular staining are listed in **Supplementary Table 1**. Fixable viability dye (eF780, eBioscience) was used for the exclusion of dead cells.

To maintain the cytoplasmic tdTom signal, cells were pre-fixed (3.5 min) using the Cytofix/Cytoperm kit (BD Biosciences) as described (12, 18, 29). For intracellular staining a second fixation/permeabilization using the Cytofix/Cytoperm kit (BD Biosciences) for a minimum of 30 min was performed followed by intracellular staining. For intracellular IFN-γ staining, cells were first stimulated as previously described (12). Samples were acquired on a BD Symphony A5 cytometer or BD Canto II flow cytometer and up to 2,000,000 events were recorded.

### Statistics and software

GraphPad Prism version 8 software (GraphPad, San Diego, CA, USA) was used for statistical analyses. To determine statistical significance between two groups Mann-Whitney test was used. To evaluate significance between multiple groups, Kruskal-Wallis test followed by corrected Dunn’s multiple comparison test was used. Differences were defined to be significant from *p* values ≤ 0.05. Radar charts were created with Excel 2019. Illustrations and figures were created with BioRender.com and Adobe Illustrator 2023. Movie editing was carried out using Adobe Premiere Pro 2023.

## Results

### Nucleofection-based CRISPR/Cas9 gene editing generates PD-1-deficient primary CD8+ T cells that are suitable for cell transfer and intravital imaging

**Figure 1:**
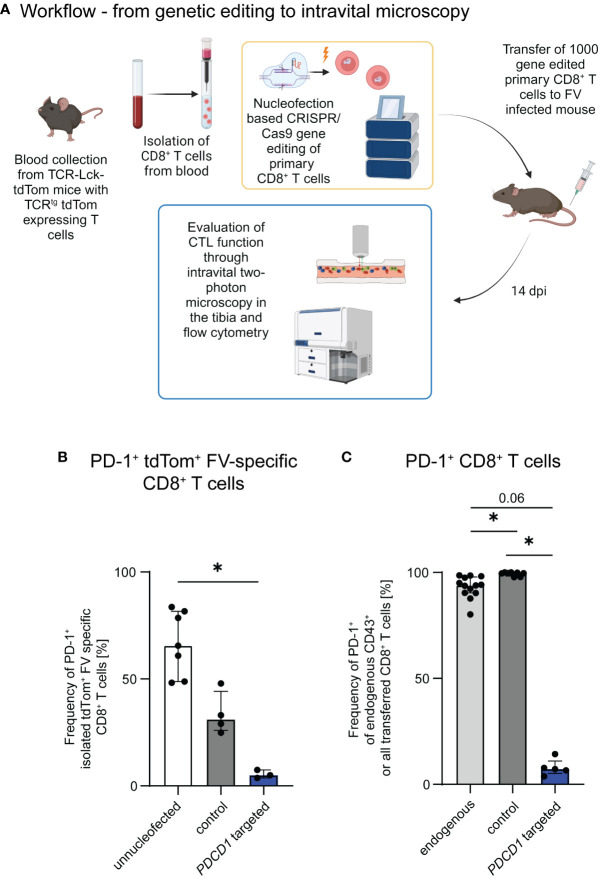
Genetic editing of primary CTLs for the evaluation of CTL motility and function. **(A)** Schematic overview of the workflow. Gene edited CTLs were generated from naïve CD8+ T cells obtained from the blood of TCR-Lck-tdTom mice using the Lonza 4D nucleofector system. C57BL/6 and DEREG recipient mice were infected with FV and subsequently received 1,000 CTLs, which were treated with gRNA/Cas9 RNPs targeting *PDCD1* or unspecific gRNA/Cas9 RNPs (control) or unnucleofected primary naïve CTLs. Intravital two-photon bone marrow microscopy and flow cytometry were performed at 14 dpi. Schematic overview was created with BioRender.com. **(B)** Frequencies of PD-1 expressing *PDCD1*-targeted, control and unnucleofected CD8+ T cells of the in-vitro KO validation (median ± IQR). Data was obtained for each single nucleofected sample in 3 independent experiments. **(C)** Frequencies of PD-1 expressing transferred or endogenous activated CTLs of FV infected mice transferred with *PDCD1*-targeted, control and unnucleofected CTLs at 14 dpi (median ± IQR). Data were obtained in 1-4 independent experiments with 1-4 mice. P-values were obtained by Kruskal-Wallis test followed by a corrected Dunn’s multiple comparison test. *p ≤ 0.05.

We have previously demonstrated, that PD-1 is upregulated on CTLs already during early stages of FV infection (15), but is associated with CTL dysfunction only in later infection stages. Immune checkpoint therapy is able to restore CTL function to a certain extent in chronic FV infection (16). We were curious, whether PD-1 expression on our transferred cells is associated with their motility. Therefore, we evaluated the PD-1 expression on our transferred FV-specific tdTom+ CTLs at 10 dpi, the time point when CTLs are at their peak of motility, and 14 dpi, when CTL motility is reduced (12). Consistent proportions of PD-1 expressing FV-specific tdTom+ CTLs were measured at both 10 dpi and 14 dpi (**Supplementary Figure 1**). However, it is known, that PD-1 expression is not restricted to exhausted cells, but upregulated on T cells upon activation, and PD-1 expressing CTLs may still provide efficient anti-viral responses (15, 30). As the PD-1-PD-L1 axis was previously shown to impair T cell motility (14), we aimed to evaluate the impact of PD-1 expression on CTL motility and effector functions in the late phase of acute FV infection. For this we adapted the protocol of Nüssing et al. (23) and Seki et al. (24) for our established intravital bone marrow imaging protocol (12, 18). We formed RNP complexes using three different guide RNAs targeting the *PDCD1* gene and nucleofected freshly isolated naive tdTom+ FV-specific CD8+ T cells with a mix of these three complexes (23, 24). To evaluate the impact of nucleofection itself without specific gene targeting we also nucleofected naive FV-specific tdTom+ CD8+ T cells with an unspecific gRNA/Cas9 RNP complex and transferred them into FV infected mice as a control group. Our approach enabled the adoptive transfer of gene-edited naïve primary CD8+ T cells into hosts within a few hours after FV infection (**Figure 1A**). The transferred cells recognize their cognate antigen, become activated and proliferate together with the endogenous FV-specific CTLs of the recipient (18). We then performed intravital two-photon microscopy (12, 18, 26–28) at 14 days post FV infection to visualize individual moving CTL in the bone marrow of hosts. We chose this time point because we previously described that CTL exhaustion and impairment of CTL motility start at 14 days post FV infection (12). To also evaluate the expression of cytotoxic molecules of CTLs we subsequently performed flow cytometry. To validate the efficiency of PD-1 KO in the transferred primary cells, we cultured a fracture of the freshly edited cells and activated them with anti-CD3 and anti-CD28 *in vitro*. This induced PD-1 expression on approximately 65% of unnucleofected control CD8+ T cells and 31% of CD8+ T cells which were nucleofected with unspecific RNP (control) within one day of culture. In contrast, only ~5% of *PDCD1* targeted cells expressed PD-1 after one day of culture (**Figure 1B**), thus showing efficient genetic manipulation. Notably, nucleofection itself appeared to impair the expression of PD-1 within 24 hours. At this time span, cells might still be under stress due to the nucleofection, which might impair the activation of the cells. To assess, whether this is a transient effect, we additionally validated PD-1 molecule expression on *PDCD1* targeted transferred versus endogenous cells at 14 days post FV infection *in vivo*. At this time point a median of 94% activated endogenous CD8+ cells and 99% of control CTLs (unspecifically nucleofected) expressed PD-1, whereas this was found only in ~7% of *PDCD1* targeted transferred cells (**Figure 1C**). Representative contour plots for **Figures 1B, C** can be found in **Supplementary Figures 2A, B**. The data demonstrated the stability and specificity of the PD-1 KO *in vivo* in T cells that recognized their cognate antigen, became activated and strongly proliferated. Hence, the used nucleofection protocol is suitable for functional gene KO in naïve primary murine CD8+ T cells followed by the adoptive transfer and intravital imaging of these cells in virus-infected hosts.

### PD-1 KO improves CTL motility and functionality in FV infection

**Figure 2:**
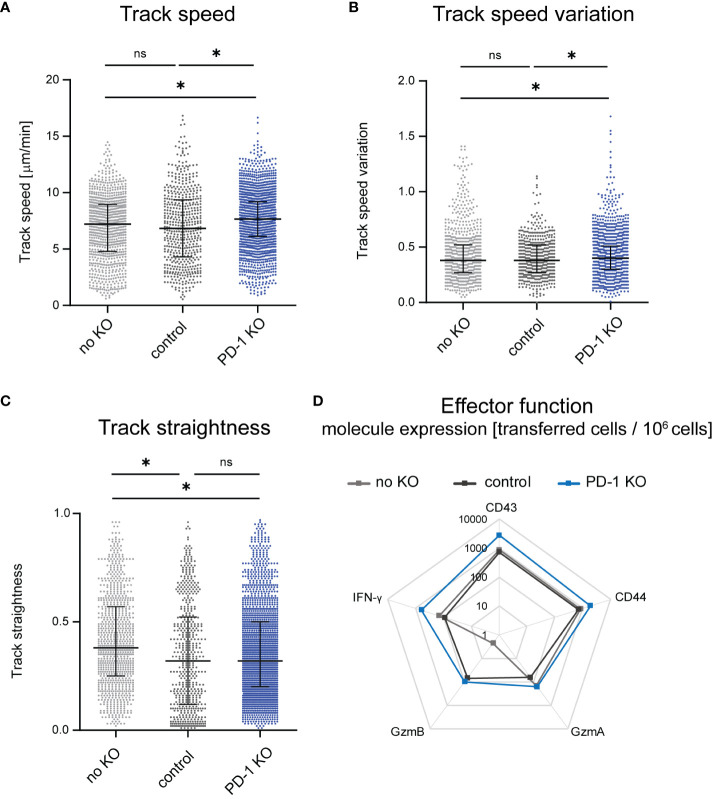
Changes of CTL motility upon PD-1 KO. Intravital two-photon microscopy in the bone marrow: CTLs were tracked in FV infected mice that received PD-1-KO CTLs, CTLs nucleofected with unspecific gRNA/Cas9 RNP (control) or unnucleofected CTLs at 14 dpi. **(A)** Mean CTL track speed [µm/min], **(B)** track speed variation of single CTLs **(C)** and CTL track straightness were analyzed from single CTL tracks. Unnnucleofected data from 3 mice was reanalyzed from previously published datasets in JCI Insight 2023 (12). Data points represent the values of single CTLs of 4 mice per group (median ± IQR) for PD-1 KO CTLs and 7 mice for control CTLs. Each mouse was imaged individually. P-values were obtained by Kruskal-Wallis test followed by a corrected Dunn’s multiple comparison test. *p ≤ 0.05. **(D)** Expression of activation- and cytotoxicity-associated molecules on transferred PD-1-KO, control or unnucleofected CTLs shown in a logarithmic scale. Flow cytometry was used to evaluate the extracellular expression of CD43 and CD44 and intracellular expression of GzmA, GzmB and IFN-γ on transferred CTLs per million cells. The mean value was calculated from one experiment with 4 mice for unnucleofected cells and 3-4 experiments with 1-2 mice for PD-1-KO CTLs and control CTLs. ns, not significant.

It was previously shown that T cell motility improves during LCMV infection upon systemic antibody blockade of the PD-1-PD-L1 axis (14). Since targeting PD-1 specifically on CD8+ T cells allows a more detailed investigation of the impact of the PD-1-PD-L1 axis on CTL motility, we used PD-1-KO CTLs to study their motility by intravital two-photon microscopy in the bone marrow. We compared the motility results to our recently published data on the motility of unmodified CTL (12), where the same principal workflow was carried out without gene editing. To exclude an effect of the nucleofection itself on the cell motility, we used a control group that received CD8+ T cells which were nucleofected with an unspecific gRNA/Cas9 RNP. We found improved CTL motility upon KO of PD-1 at 14 days post FV infection in comparison to non-edited and control nucleofected CTLs. This was reflected by an increase in the mean CTL track speed, which in control nucleofected cells was at 6.8 µm/min while the median of PD-1-KO cells was significantly increased to 7.7 µm/min (**Figure 2A**). Moreover, also the track speed variation (track standard deviation divided by track speed mean) increased slightly in PD-1-KO CTL (**Figure 2B**), which mirrors an improvement in the dynamic motility response of CTLs enabling them to speed up and slow down throughout their track. When analyzing CTL track straightness (direct track distance divided by track length), we found comparable levels in PD-1 KO CTLs compared to control nucleofected CTLs. In both groups, the track straightness was reduced compared to unnucleofected CTLs, indicating, that nucleofected CTLs had more random movement with more frequent directional changes, possibly towards target cells (**Figure 2C**).

For the evaluation of effector functions we stained CTL for several activation and cytotoxicity markers after isolating cells at 14 days post FV infection (and 14 days post adoptive transfer) using flow cytometry as read out and compared transferred PD-1-KO CTLs with control nucleofected and non-edited transferred CTLs. Exemplary pseudocolour dot plots can be found in **Supplementary Figure 3**. These datasets revealed enhanced expression of the activation markers CD43 and CD44 on PD-1-KO CTLs compared to control nucleofected and unnucleofected transferred cells, indicating that *in vivo* T cell activation was strong in PD-1-KO cells. While no differences were detected concerning the numbers of Granzyme (Gzm) A positive PD-1 KO CTLs to unnucleofected CTLs were found, a slight increase of the number of GzmA+ PD-1 KO CTLs compared to control nucleofected CTLs was observed. Furthermore, we found increased numbers of GzmB positive PD-1-KO CTL and control nucleofected CTLs compared to non-edited transferred cells. Of note, the difference in the amount of GzmA and GzmB expressing CTLs was based on very low overall numbers. Moreover, we found an increased number of Interferon-γ (IFN-γ) expressing CTLs when PD-1 was removed compared to control nucleofected and unnucleofected cells (**Figure 2D**), indicating that PD-1 KO resulted in augmented CTL functional properties. Overall, the KO of PD-1 improved CTL motility and activation as well as effector molecule expression *in vivo*. To evaluate, whether the improved cytotoxic molecule expression takes an impact on the anti-viral response we evaluated virus titers in mice, which received PD-1 KO, control-nucleofected or unnucleofected CTLs. Virus titers were similar between the different groups, indicating that, not surprisingly, the transferred amount of 1,000 edited CTLs is not sufficient to dampen the viral load (**Supplementary Figure 4**).

## Discussion

In this study we adapted a previously published CRISPR/Cas9 based approach to edit naïve primary CTLs (23). We used a combination of up to three different gRNA/Cas9-RNPs for the KO of the same gene (24). We chose this approach over the systemic therapy with anti-PD-1 or anti-PD-L1 blocking antibodies. Systemic therapy changes the overall immune response of the host, as it interacts with all cell types expressing PD-1 or PD-L1, which can result in severe bystander effects on CTL motility and function. Targeting CTL attributes the observed effect directly to the cell type of interest. This approach is also very interesting for cell therapy approaches, like Chimeric Antigen Receptor (CAR)-T-cell therapy (31). We have previously shown that FV-specific CTL (naïve TCR transgenic FV-specific CD8+ T cells) become activated in chronically FV-infected mice, because they recognize their cognate antigen. However, they become very rapidly functionally exhausted because of the overall suppressive environment during chronic infection (32, 33). The editing with CRISPR/Cas9 technology of primary T cells might provide a tool to genetically modify T cells and prevent the development of exhaustion during therapy.

In this study we showed that expression of PD-1 on CTLs decreases their motility *in vivo* and that KO of PD-1 can be used to improve CTL motility and other functional properties. In studies using the persistent CL13 LCMV strain, systemic therapy with anti-PD-1 blocking antibodies also improved CTL motility. The authors described that the PD-1-PD-L1 axis promotes stable immunological synapse formation rather than the formation of instable kinapses in a lipid bilayer model. Moreover, the block of the PD-1-PD-L1 interaction on CTLs improves their cell signaling and their motility (14). Changes in CTL motility were more prominent during LCMV infection upon systemic blockage of the PD-1-PD-L1 axis (14) compared to our findings with PD-1-KO CTLs during FV infection. On the one hand these results validate each other, but also suggest that additional bystander effects are active after systemic antibody blocking therapy and the effect may vary upon different infection models. Together they identify PD-1 as an important target for improving CTL motility in virus infections (14). Of note, in our studies CTL motility was not completely restored upon PD-1 KO to levels of the peak CTL response, where CTLs move with a median speed of ~8 µm/min (12). Furthermore, when comparing CTL motility of PD-1-KO CTLs to motility in DEREG mice, which were treated with diphtheria toxin for depletion of Tregs, PD-1-KO CTLs did also not reach this level of motility (12). This indicates that both, the inhibitory receptor PD-1 as well as Tregs, inhibit CTL motility *in vivo* and that these mechanisms seem to synergize. Previous studies of our group showed, that PD-L1 together with Tim-3 blocking antibody therapy was more effective in reactivating exhausted CTLs in chronic FV infection compared to depletion of Tregs, but the combination of both was superior compared to any single therapy (16). This is in line with our conclusion that both pathways negatively influence CTL motility and functional properties of CTL and significantly contribute to CTL exhaustion. Of note, the combination therapy of immune checkpoint blocking antibodies and depletion of Tregs led to lethal immunopathology in acute FV infection, highlighting the delicate balance of pro-inflammatory and counter-regulatory immune responses in infectious diseases (34). Thus, adoptive transfer of gene-targeted cells is a more precise approach compared to a broad systemic therapy and might also be useful in novel immunotherapies against viruses.

To conclude, our adapted gene editing protocol enables the targeting of genes in primary naïve CTLs and is suitable for adoptive cell transfer experiments and a subsequent visualization of CTL motility through intravital two-photon microscopy multiple days after virus infection and cell transfer. Targeting *PDCD1* led to improved CTL motility, which now can be directly attributed to ligand interaction with PD-1 on CTLs. This gene-editing protocol is not limited to the KO of PD-1 but can also be used to target any other gene that is important for CTL motility and function. Hence, this approach might be relevant to improve therapeutic potential of T cells for example in CAR-T cell therapy.

## Data availability statement

The raw data supporting the conclusions of this article will be made available by the authors, without undue reservation.

## Ethics statement

The animal study was approved by Landesamt für Natur, Umwelt und Verbraucherschutz Nordrhein-Westfalen (LANUV). The study was conducted in accordance with the local legislation and institutional requirements.

## Author contributions

DM: Conceptualization, Formal analysis, Investigation, Methodology, Visualization, Writing – original draft, Writing – review & editing. LO: Investigation, Methodology, Supervision, Writing – review & editing, Conceptualization, Formal analysis. AK: Methodology, Supervision, Writing – review & editing. A-KS: Methodology, Writing – review & editing. EL-S: Methodology, Writing – review & editing. AH: Methodology, Writing – review & editing, Supervision. UD: Funding acquisition, Methodology, Project administration, Resources, Supervision, Writing – review & editing, Conceptualization. MG: Funding acquisition, Project administration, Resources, Supervision, Writing – review & editing, Conceptualization.

## Supplementary material

Supplementary Figure 1 PD-1 expression of transferred CTLs in FV infection. Percentage of PD-1 expressing transferred tdTomato+ FV-specific CTLs was determined in FV infected C57BL/6 mice at 10 and 14 dpi using flow cytometry (median ± IQR). Data was obtained from one experiment with 4 mice for each group. Statistical significance was tested using the Mann-Whitney test.

Supplementary Figure 2 Validation of PD-1 knockout. Expression of PD-1 was determined *in vitro* and *in vivo* using flow cytometry. **(A)** PD-1 expression *in vitro* was determined 1-day post activation on CD8+ T cells nucleofected with PDCD1 targeting gRNA/Cas9 RNP, unspecific gRNA/Cas9 RNP (control) or left unnucleofected. **(B)** *In vivo* PD-1 expression was determined on endogenous, control nucleofected and PD-1 KO CTLs in mice 14 dpi. Data is shown as representative contour plots. Data from all nucleofections and experimental groups can be seen in **Figures 1B, C**.

Supplementary Figure 3 Activation- and cytotoxicity-associated markers in transferred CTLs. C57BL/6 mice and DEREG mice were infected with FV and received CTLs which were treated with PDCD1 targeted or unspecific gRNA/Cas9 RNP complex (control) or left unnucleofected. The number of CD43, CD44, GzmA, GzmB and IFN-γ expressing transferred CTLs per million cells was determined at 14 dpi using flow cytometry and the calculated data is displayed in **Figure 2D**. Representative pseudocolour dot plots for each marker are displayed for unnucleofected, control-nucleofected and PD-1 KO CTLs together with the percentage of positive cells per transferred cells. Gates were set first for endogenous control cells based on unstained controls, naïve controls and control stainings lacking CD43, CD44, GzmA, GzmB and IFN-γ staining or lacking GzmA, GzmB and IFN-γ staining. From there gates were taken over for transferred cells. Gating was performed for every experiment individually to overcome technical variations between days.

Supplementary Figure 4 Virus titers in recipient mice. Virus titers were evaluated in FV infected C57BL/6 and DEREG mice which received PD-1 KO CTLs, control CTLs or unnucleofected CTLs at 14 dpi using an infectious center assay (median ± IQR). Virus titers were evaluated in 2-4 independent experiments with 1-4 mice each. Statistic differences were determined using Kruskal-Wallis test followed by a corrected Dunn’s multiple comparison test.

Supplementary Table 1 Antibodies used for extracellular and intracellular flow cytometry.

Supplementary Movie 1 Evaluation of PD-1-KO CTL motility. Intravital two-photon microscopy in the tibial bone marrow: representative video of tracking FV-specific tdTom+ PD-1-KO CTLs in FV infected mice at 14 dpi. Cyan: *PDCD1* targeted CTLs, yellow tracks: PD-1-KO CTL tracks. Cells were automatically tracked using the IMARIS spot function and spots are indicated through yellow surrounding. CTL tracks are indicated using dragon tails for a continuous time span of 5 min.
