# Supplementary figures and images for: PD-1 knockout on cytotoxic primary murine CD8+ T cells improves their motility in retrovirus infected mice
Source: Front Immunol. 2024 Apr 29;15:1338218. doi: 10.3389/fimmu.2024.1338218 (PMC11089113; doi:10.3389/fimmu.2024.1338218)

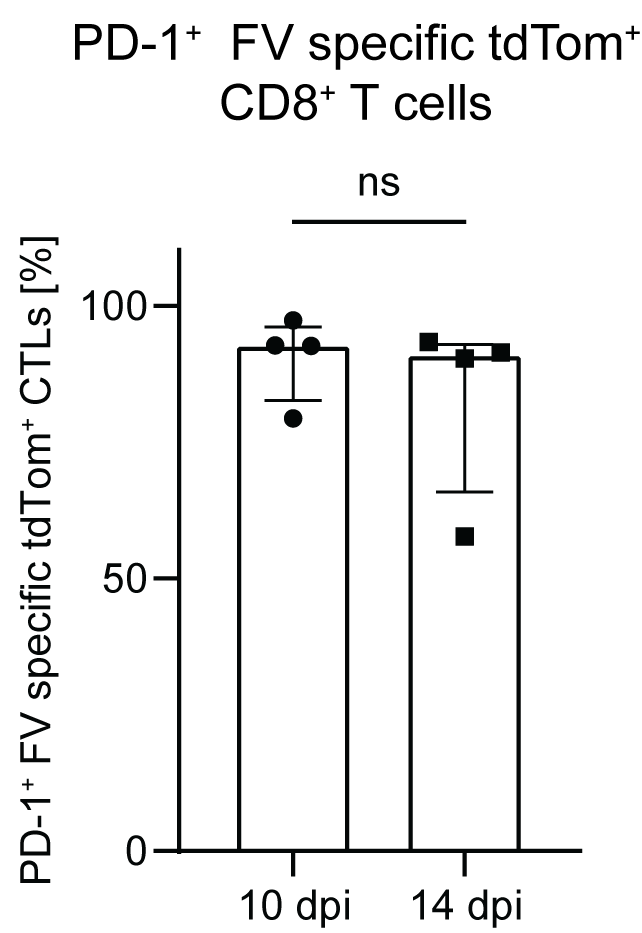

Supplement: Supplementary Figure 1 — PD-1 expression of transferred CTLs in FV infection. Percentage of PD-1 expressing transferred tdTomato+ FV-specific CTLs was determined in FV infected C57BL/6 mice at 10 and 14 dpi using flow cytometry (median ± IQR). Data was obtained from one experiment with 4 mice for each group. Statistical significance was tested using the Mann-Whitney test. [file Image_1.tif]

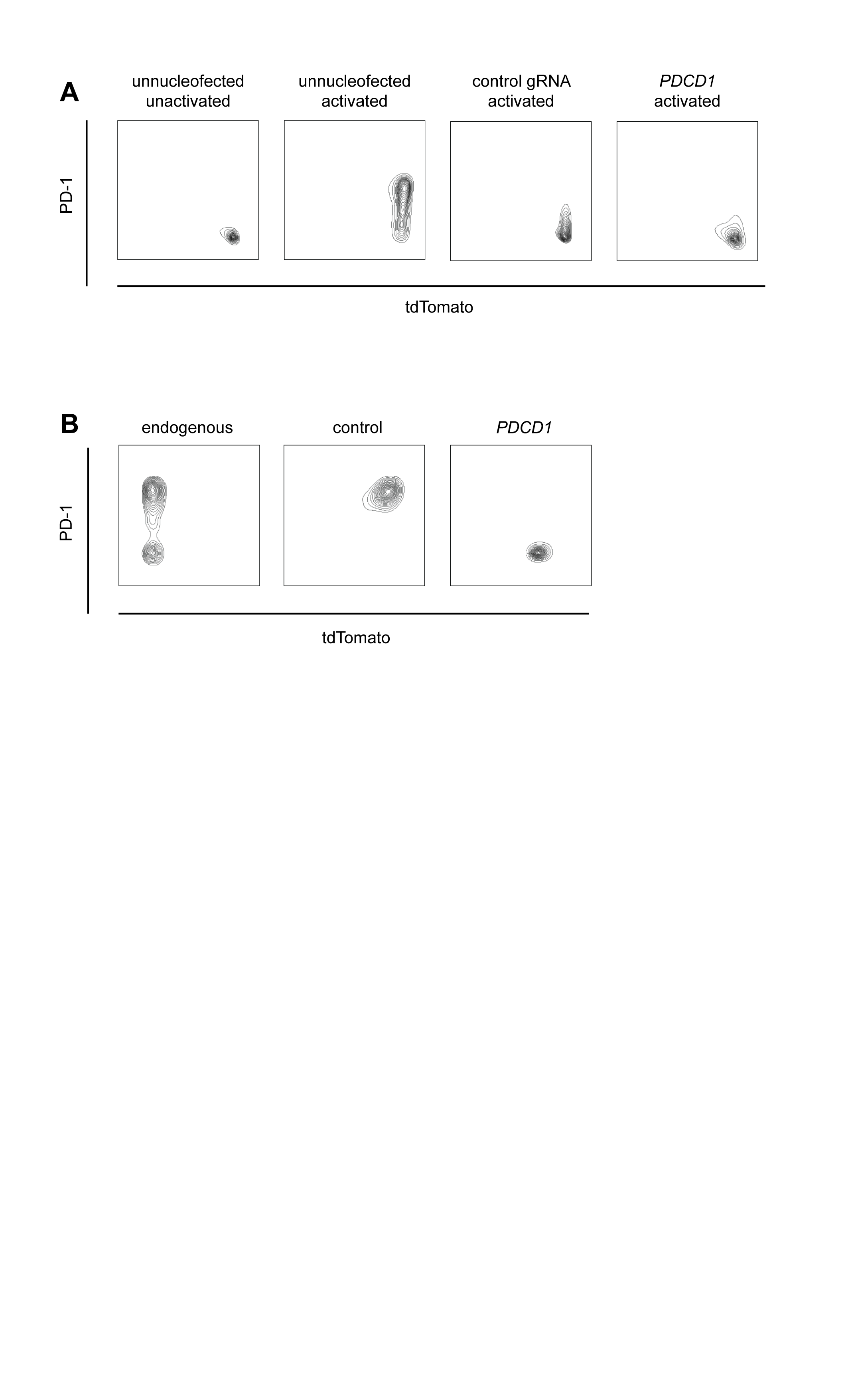

Supplement: Supplementary Figure 2 — Validation of PD-1 knockout. Expression of PD-1 was determined in vitro and in vivo using flow cytometry. (A) PD-1 expression in vitro was determined 1-day post activation on CD8+ T cells nucleofected with PDCD1 targeting gRNA/Cas9 RNP, unspecific gRNA/Cas9 RNP (control) or left unnucleofected. (B) In vivo PD-1 expression was determined on endogenous, control nucleofected and PD-1 KO CTLs in mice 14 dpi. Data is shown as representative contour plots. Data from all nucleofections and experimental groups can be seen in Figures 1B, C . [file Image_2.tif]

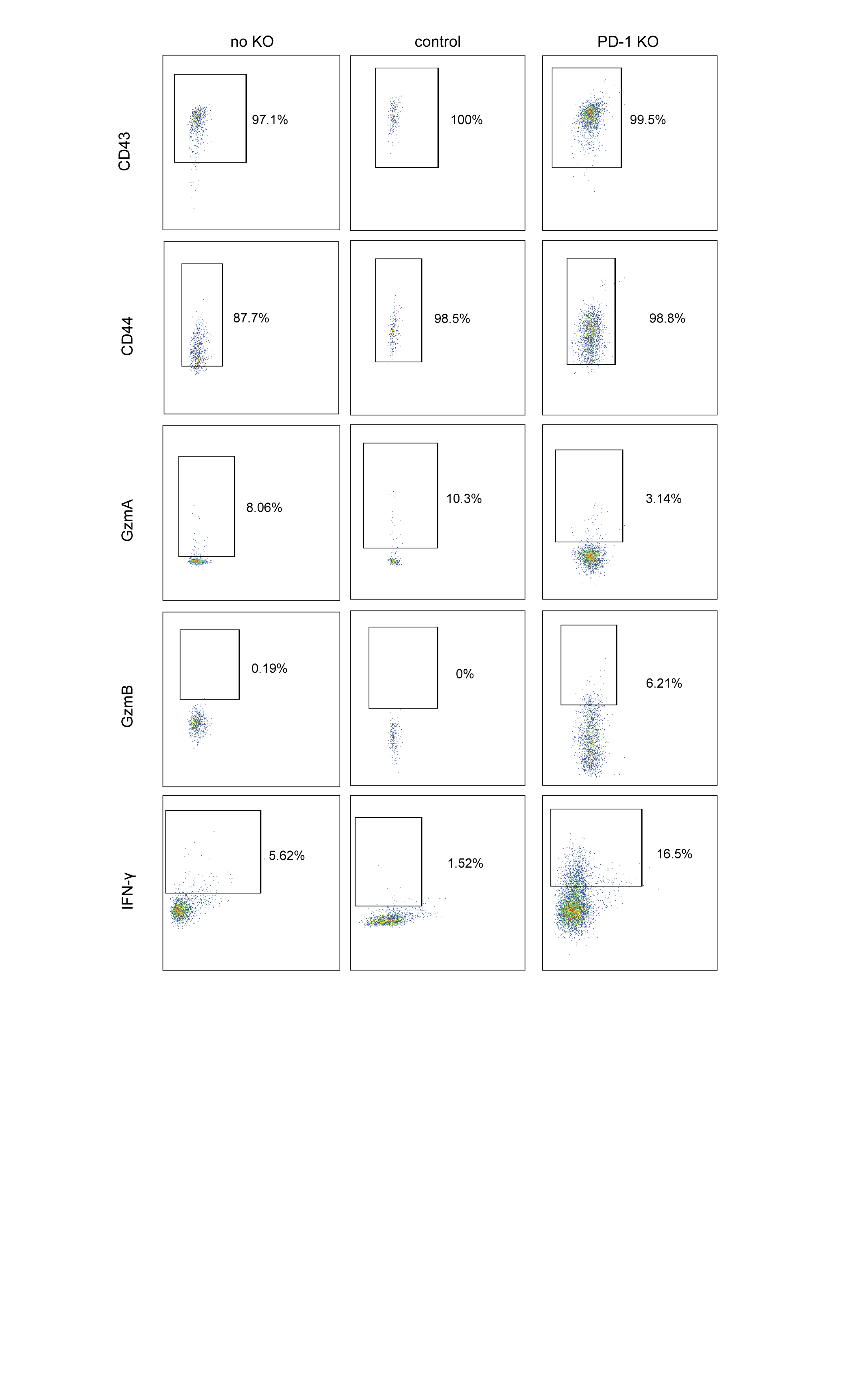

Supplement: Supplementary Figure 3 — Activation- and cytotoxicity-associated markers in transferred CTLs. C57BL/6 mice and DEREG mice were infected with FV and received CTLs which were treated with PDCD1 targeted or unspecific gRNA/Cas9 RNP complex (control) or left unnucleofected. The number of CD43, CD44, GzmA, GzmB and IFN-γ expressing transferred CTLs per million cells was determined at 14 dpi using flow cytometry and the calculated data is displayed in Figure 2D . Representative pseudocolour dot plots for each marker are displayed for unnucleofected, control-nucleofected and PD-1 KO CTLs together with the percentage of positive cells per transferred cells. Gates were set first for endogenous control cells based on unstained controls, naïve controls and control stainings lacking CD43, CD44, GzmA, GzmB and IFN-γ staining or lacking GzmA, GzmB and IFN-γ staining. From there gates were taken over for transferred cells. Gating was performed for every experiment individually to overcome technical variations between days. [file Image_3.tif]

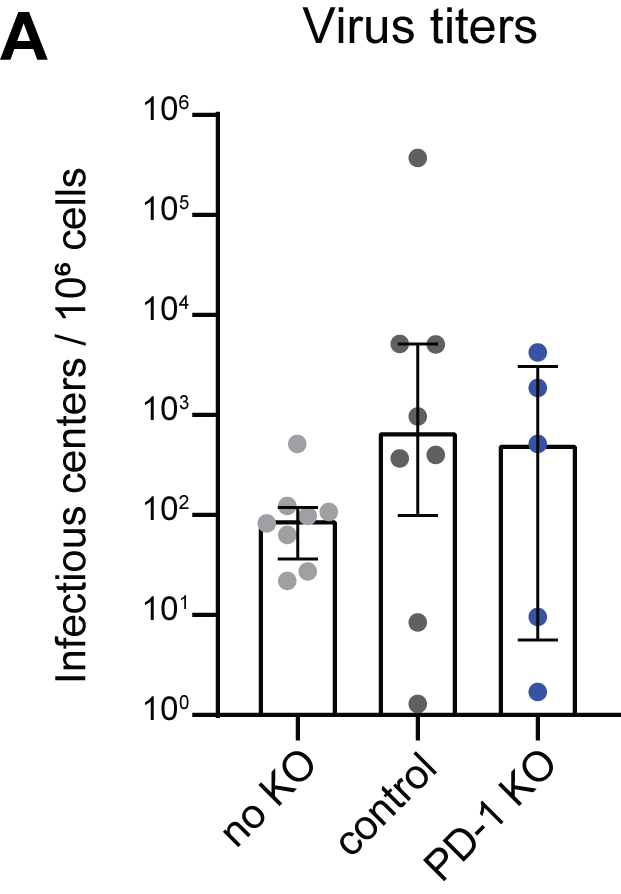

Supplement: Supplementary Figure 4 — Virus titers in recipient mice. Virus titers were evaluated in FV infected C57BL/6 and DEREG mice which received PD-1 KO CTLs, control CTLs or unnucleofected CTLs at 14 dpi using an infectious center assay (median ± IQR). Virus titers were evaluated in 2-4 independent experiments with 1-4 mice each. Statistic differences were determined using Kruskal-Wallis test followed by a corrected Dunn’s multiple comparison test. [file Image_4.tif]
